# Parallel learning and cognitive flexibility impairments between *Fmr1* knockout mice and individuals with fragile X syndrome

**DOI:** 10.3389/fnbeh.2022.1074682

**Published:** 2023-01-05

**Authors:** Lauren M. Schmitt, Anna L. Arzuaga, Ashley Dapore, Jason Duncan, Maya Patel, John R. Larson, Craig A. Erickson, John A. Sweeney, Michael E. Ragozzino

**Affiliations:** ^1^Division of Behavioral Medicine and Clinical Psychology, Cincinnati Children’s Hospital Medical Center, Cincinnati, OH, United States; ^2^Department of Pediatrics, University of Cincinnati College of Medicine, Cincinnati, OH, United States; ^3^Department of Psychology, University of Illinois Chicago, Chicago, IL, United States; ^4^Department of Psychiatry, Cincinnati Children’s Hospital Medical Center, Cincinnati, OH, United States; ^5^Department of Psychiatry, University of Illinois Chicago, Chicago, IL, United States; ^6^Department of Psychiatry and Behavioral Neuroscience, University of Cincinnati College of Medicine, Cincinnati, OH, United States

**Keywords:** fragile X syndrome, *FMR1*, cognitive flexibility, executive function, autism

## Abstract

**Introduction:**

Fragile X Syndrome (FXS) is a monogenic condition that leads to intellectual disability along with behavioral and learning difficulties. Among behavioral and learning difficulties, cognitive flexibility impairments are among the most commonly reported in FXS, which significantly impacts daily living. Despite the extensive use of the *Fmr1* knockout (KO) mouse to understand molecular, synaptic and behavioral alterations related to FXS, there has been limited development of translational paradigms to understand cognitive flexibility that can be employed in both animal models and individuals with FXS to facilitate treatment development.

**Methods:**

To begin addressing this limitation, a parallel set of studies were carried out that investigated probabilistic reversal learning along with other behavioral and cognitive tests in individuals with FXS and *Fmr1* KO mice. Fifty-five adolescents and adults with FXS (67% male) and 34 age- and sex-matched typically developing controls (62% male) completed an initial probabilistic learning training task and a probabilistic reversal learning task.

**Results:**

In males with FXS, both initial probabilistic learning and reversal learning deficits were found. However, in females with FXS, we only observed reversal learning deficits. Reversal learning deficits related to more severe psychiatric features in females with FXS, whereas increased sensitivity to negative feedback (lose:shift errors) unexpectedly appear to be adaptive in males with FXS. Male *Fmr1* KO mice exhibited both an initial probabilistic learning and reversal learning deficit compared to that of wildtype (WT) mice. Female *Fmr1* KO mice were selectively impaired on probabilistic reversal learning. In a prepotent response inhibition test, both male and female *Fmr1* KO mice were impaired in learning to choose a non-preferred spatial location to receive a food reward compared to that of WT mice. Neither male nor female *Fmr1* KO mice exhibited a change in anxiety compared to that of WT mice.

**Discussion:**

Together, our findings demonstrate strikingly similar sex-dependent learning disturbances across individuals with FXS and *Fmr1* KO mice. This suggests the promise of using analogous paradigms of cognitive flexibility across species that may speed treatment development to improve lives of individuals with FXS.

## 1. Introduction

Fragile X Syndrome (FXS) is the leading single gene cause of inherited intellectual disability and autism spectrum disorder (ASD) (Turner et al., 1996), and is caused by a cytosine-guanine-guanine (CGG) trinucleotide repeat expansion in the promoter region of the fragile X messenger ribonucleoprotein 1 (*FMR1*) gene (Fu et al., 1991). Expansions of 200 or more CGG repeats result in hyper-methylation and silencing of the *FMR1* gene, leading to lost expression of fragile X messenger ribonucleoprotein (FMRP). Because FMRP is critical for synaptic structure and function (Pieretti et al., 1991; Park et al., 2008; Kao and Liu, 2010), the absence of FMRP is associated with atypical brain development and characteristic phenotypic features, including impaired cognitive development (Antar and Bassell, 2003; Bassell and Warren, 2008).

Deficits in cognitive flexibility are frequent, pronounced, and among the most functionally impairing behavioral features in FXS (Weber et al., 2019), often manifesting themselves as repetitive behaviors including perseverative questioning and rigid behavioral preferences (Schmitt et al., 2019b; Reisinger et al., 2020). Cognitive flexibility impairments can significantly limit daily living, independence and positive social relations across a range of disorders (Delahunty et al., 1993; Razani et al., 2007; Amorim and Marques, 2018; Oracki, 2021). Conversely, under certain conditions cognitive flexibility training can improve daily living skills (Delahunty et al., 1993). Thus, developing treatments that improve cognitive flexibility in FXS individuals may produce significant benefits to enhance their quality of life.

Despite cognitive flexibility impairments commonly reported in FXS individuals (Hooper et al., 2008, 2018; Schmitt et al., 2019b; Weber et al., 2019), there is limited understanding of what underlying cognitive processes are altered that contribute to a cognitive flexibility deficit in FXS. This is critical to identify as numerous preclinical studies in rodents have shown that pharmacological treatments that improve cognitive flexibility, i.e., set-shifting or reversal learning, modify specific processes by either reducing perseveration of a previously learned strategy or increasing sensitivity to positive reinforcement (Boulougouris et al., 2008; Brown et al., 2012; Phillips et al., 2018; Dunn et al., 2020; Wilkinson et al., 2020; Athnaiel et al., 2022). In addition, while the absence of FMRP has been modeled in the *Fmr1*-knockout (KO) mouse, which can display behavioral flexibility deficits (Van Dam et al., 2005; McNaughton et al., 2008; Casten et al., 2011; Dickson et al., 2013; Kazdoba et al., 2014; Nolan and Lugo, 2018; Vershkov et al., 2022), a further challenge is developing cognitive flexibility tests that have a significant translational component so that more direct comparisons can occur between clinical and preclinical findings, as well as improving the probability that treatments found effective in preclinical experiments successfully translate to the clinic.

One paradigm that has been successfully employed to investigate cognitive flexibility in preclinical and clinical studies is a probabilistic reversal learning test (Peterson et al., 2009; Amodeo et al., 2012, 2017; D’Cruz et al., 2013; Robbins, 2017; Grospe et al., 2018; Schmitt et al., 2019a,^2022^). The test has been used in studies with humans, non-human primates and rodents to identify whether a reversal learning deficit results from initial perseveration of the previously learned choice pattern and/or from maintaining the new choice pattern after initial selection. Furthermore, the paradigm has allowed detecting whether a reversal learning deficit emerges due to an altered sensitivity to positive reinforcement and/or due to negative reinforcement. Moreover, the probabilistic reversal learning test has been used in preclinical and clinical studies to determine what processes pharmacological and behavioral intervention treatments affect to influence cognitive flexibility (Bari et al., 2010; Brown et al., 2012; Skandali et al., 2018; Schmitt et al., 2022). Thus, probabilistic reversal learning represents one test that could be used as a cross-species paradigm related to FXS to identify altered cognitive processes underlying cognitive inflexibility and develop treatments that effectively translate to positive clinical outcomes.

In taking a cross-species translational approach, the present experiments sought to identify whether FXS individuals and *Fmr1*-KO mice exhibit similar probabilistic learning and/or reversal learning deficits and if so, whether deficits share underlying processes in both species. In individuals with FXS, there was a further determination of whether performance measures in the probabilistic learning and reversal tests related to broader cognitive, behavioral, and psychiatric features. In *Fmr1*-KO mice, to further characterize possible cognitive flexibility impairments, wildtype (WT) and *Fmr1*-KO mice were tested in the reward conflict test (Brown et al., 2012) to determine how *Fmr1*-KO mice perform when required to inhibit a prepotent biased response pattern.

## 2. Materials and methods

### 2.1. Subjects

#### 2.1.1. Humans

Fifty-five participants with a genetic diagnosis of FXS (5–47 years, 67% male) and 34 age- and sex-matched controls (4–45 years, 62% male) completed testing (Table 1). Participants with FXS were recruited from existing patients at the Cincinnati Fragile X Research and Treatment Center, postings on the National Fragile X Foundation website, and at conferences such as the International Fragile X Conference. Fragile X diagnosis was confirmed *via* polymerase chain reaction (PCR) and/or Southern Blot assays at Rush University in the laboratory of Dr. Elizabeth Berry-Kravis. Sixteen males were fully methylated, full mutation, 10 males were either size and/or methylation mosaicism, and 11 males did not have Southern Blot assays available to confirm mosaicism status. Control participants typically developing control (TDC) were recruited *via* flyers posted in Cincinnati Children’s Hospital Medical Center and in the community, and were required to have no personal history of psychiatric or neurologic disorders, no first- or second-degree relatives with ASD or other familial neuropsychiatric illness, and a Social Communication Questionnaire score ≤8.

**TABLE 1:** Demographic characteristics of fragile X syndrome (FXS) and TDC participants.

	FXS Male (*n* = 37)	FXS Female (*n* = 18)	FXS (*n* = 55)	TDC Male (*n* = 21)	TDC Female (*n* = 13)	TDC (*n* = 34)
Age	25.1 (10.3)	24.6 (11.2)	24.9 (10.5)	21.7 (10.4)	26.3 (10.8)	23.5 (10.6)
Deviation IQ	31.4 (20.8)***^∧^	71.3 (29.7)**	44.7 (30.5)***	108.9 (10.8)	99.0 (11.2)	105.1 (11.8)
Non-verbal Z-score	−5.4 (1.9)***^∧^	−2.4 (2.4)**	−4.4 (2.5)***	0.6 (0.8)	−0.2 (1.2)	0.3 (1.0)
Verbal Z-score	−3.8 (1.3)***^∧^	−1.5 (1.7)**	−3.0 (1.8)***	0.6 (0.8)	0.1 (0.7)	0.4 (0.8)
SCQ	16.2 (8.1)	7.9 (6.0)	13.4 (8.4)			

Mean (SD) given unless otherwise indicated; ***p < 0.001, **p < 0.01 comparisons against same-sex counterpart; ^∧^*p* < 0.001 comparison against FXS male versus FXS female.

All participants completed the Abbreviated Stanford Binet-5th edition. Standard scores were converted to Deviation IQ scores and scaled scores were converted to z-scores in order to reduce floor effects present for individuals with severe cognitive impairments and to best estimate intellectual ability (Sansone et al., 2014). Primary caregivers of participants with FXS completed a battery of outcome measures, including the Aberrant Behavior Checklist-Community [ABC; (Aman et al., 1985)], Social Communication Questionnaire–Lifetime [SCQ; (Rutter et al., 2003)], and Social Responsiveness Scale [SRS-II; (Constantino, 2011)], Repetitive Behavior Scale-R [RBS-R; (Bodfish et al., 1999)], and Sensory Profile (Dunn, 2014). Many FXS participants were being treated with psychotropic medications at the time of testing, and had been on stable dosing for at least 4 weeks (Supplementary Table 1). No control participant was taking any psychotropic medications within at least 4 weeks of testing.

All participants, or their legal guardian when appropriate, provided written informed consent and assent before completing research activities. Study procedures were approved by the Institutional Review Board at Cincinnati Children’s Hospital.

#### 2.1.2. Mice

The mouse experiments were performed by breeding *Fmr1*-KO or WT male and *Fmr1* heterozygous (HET) females in the laboratory to generate the tested male and female offspring. Initial breeders (B6.129P2-*Fmr1*^*TM*1Cgr^/J, strain #: 003025, were acquired from the Jackson Laboratory, Bar Harbor, ME). Mice were individually housed in plastic cages (28 cm wide × 17 cm long × 12 cm high) in a humidity (32% and temperature (23°C) controlled room and were maintained on a 12 h light/dark cycle (lights on at 7:30 a.m.). Mice were 8–12 weeks of age at the time of behavioral testing. Separate mice were tested in the probabilistic reversal learning test than those tested in the elevated plus maze and reward conflict test. Both heterozygous and homozygous *Fmr1* female mice were tested. Because there was no difference in the behavioral results between the different female genotypes they were combined into a single group for analyses. Animal care and experiments were followed in accordance with the National Institute of Health Guide for the Care and Use of Laboratory Animals and were approved by the Institutional Laboratory Animal Care and Use Committee of the University of Illinois Chicago. This included providing a power analysis for all behavioral tests. For example, in the spatial discrimination test with probabilistic reinforcement, the power analysis indicated to detect a difference of 15 trials or more among the groups, assuming a standard deviation of 5 in all groups at a 5% significance level and 80% power a sample of 12 would be required in each group.

### 2.2. Human testing

#### 2.2.1. Reversal learning and probabilistic training

Participants completed a practice test to ensure task comprehension. Prior to the practice test, participants watched a video demonstration which was paired with scripted verbal instructions. Instructions were modified according to participant’s receptive language abilities, as needed. Participants were presented with two identical stimuli (animal pictures) positioned on different locations on a computer screen (D’Cruz et al., 2013; Schmitt et al., 2019a,^2022^). Participants were required to select the stimuli that was in the correct location, which was reinforced with cartoon coins. Participants made their stimulus choice on a button box.

During the training task, participant behavior was reinforced on 80% of correct responses and on 20% of incorrect responses. However, no reversal learning was present as the training task was meant to ensure comprehension of the probabilistic reinforcement feedback. Participants had 100 trials to reach the criterion of 8 out of 10 consecutive correct responses. If participants failed to reach this criterion, they did not advance to the reversal learning phase. Three males (two full mutation) and one female with FXS failed the training task (age range 29–42 years, IQ range −10–38), and thus a total of 51 participants with FXS and 34 controls were completed the probabilistic reversal learning task.

#### 2.2.2. Probabilistic reversal learning task

**FIGURE 1:**
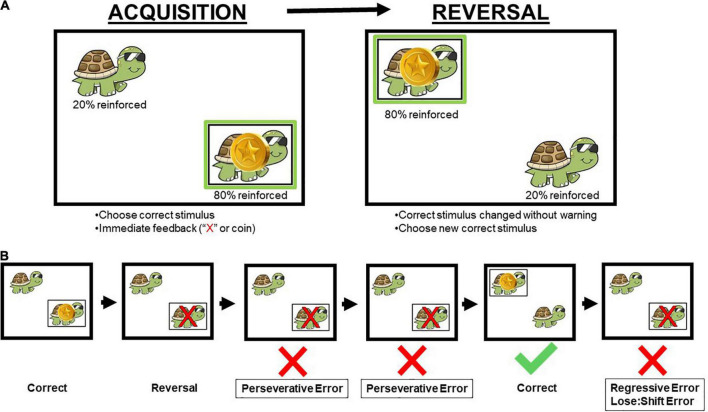
Schematic of the human probabilistic reversal learning task **(A)** and error types **(B)**. Participants were instructed to select the animal in the correct stimulus location on the screen (Acquisition). Once participants reached performance criterion, the correct stimulus location changed without warning to the other location on the screen (Reversal). Across both acquisition and reversal phases, reinforcement was provided on an 80:20 probabilistic schedule, and correct responses were reinforced with coins. A red “X” appeared over the animal if the incorrect stimulus location was chosen by the participant. Perseverative errors occurred when a participant chose the previously reinforced location prior to choosing the newly reinforced (correct) location. Regressive errors occurred when a participant chose the previously reinforced location after choosing the newly reinforced location. Lose:shift errors are a specific type of regressive error when participants chose the previously reinforced location immediately following inaccurate feedback (e.g., 20% of trials) that their choice was incorrect. Animal and location on the screen were randomized for each participant with a total of four possible combinations of animal and location. Permission to use image from author (Schmitt et al., 2022).

Participants were given the instructions “Find the animal that is usually in the correct location, and to choose that animal every time. After a while the correct location may change, choose the animal in the new correct location.” During the acquisition phase, participants must have identified the correct location on 8 of 10 consecutive trials to proceed to the reversal phase in which the correct location is switched without warning, and participants had to identify the new correct location on 8 of 10 consecutive trials. If participants did not reach criterion within 50 trials on either phase, testing was discontinued. Six individuals with FXS did not reach criterion. Consistent with the training task, participant behavior was reinforced on 80% of correct responses and on 20% of incorrect responses during both acquisition and reversal phases (Figure 1).

We computed the total number of trials to achieve the learning criterion during both the acquisition and reversal learning phases of the task. During the probabilistic reversal learning (PRL) phase, we computed the total number of errors as well as number of perseverative errors (i.e., continuing to choose the previously correct response following a change in location of the correct stimulus) and regressive errors (i.e., returning to the previously correct location after selecting the new correct location after reversal). Last, number of lose-shift errors also were computed during both the acquisition and reversal learning phases of the PRL task. When a previously correct response is not reinforced, the participant may incorrectly continue to select the same response and not receive reinforcement; this is termed a lose-shift error. These were calculated for both the acquisition and reversal phases of the PRL task.

#### 2.2.3. Other tasks of executive function

Participants completed the computerized Kiddie Test of Attentional Performance [KiTAP; (Testsysteme, 2011)], which examines multiple aspects of executive functioning over multiple domains, including processing speed, distractibility, cognitive flexibility, and behavioral response inhibition. Raw errors scores were used for statistical analysis. KiTAP has been validated for use in adults with full mutation FXS (Knox et al., 2012).

### 2.3. Mouse testing

#### 2.3.1. Spatial discrimination training

**FIGURE 2:**
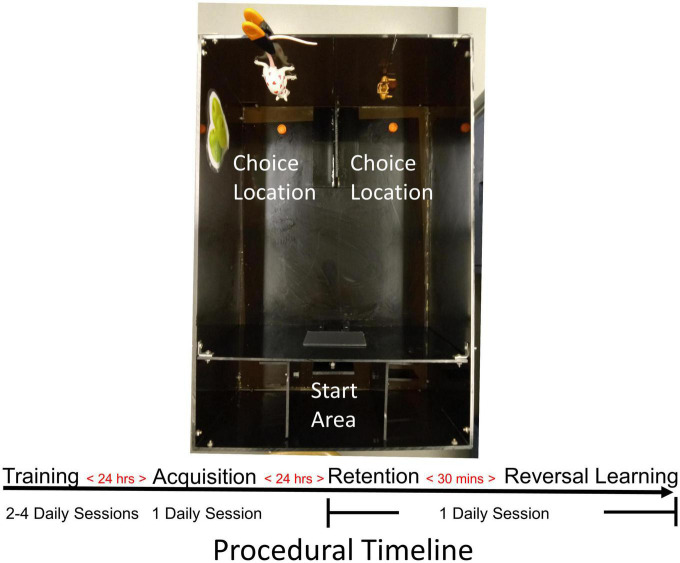
Mouse probabilistic reversal learning task. Photograph of the apparatus used for testing mice in the acquisition and reversal learning of a spatial discrimination with probabilistic reinforcement. Food-restricted mice were first trained to recover sweetened cereal pieces from food wells in choice areas. After achieving criterion, mice received an acquisition test the following day. The day after acquisition, mice first received a retention test followed 30 min later by a reversal learning test. In all tests, a 80/20 probabilistic reinforcement schedule was used such that the “correct” choice was reinforced with 80% probability.

For the probabilistic learning and reversal learning test, each mouse had free access to water in their home cage throughout the study. Mice were food restricted and maintained at 85% of their *ad libitum* body weight. Reduction to 85% of *ad libitum* body weight occurred within 4–6 days. Mice were not handled extensively prior to training and testing, as the behavioral tests only required an experimenter to place a mouse in the start area at beginning of a training or test session and removal of a mouse at the end of a session. After stabilizing a mouse’s body weight, behavioral training was started and occurred 2–4 days before testing. With the exception of ALA, experimenters (JD, MP and MR) were blind to the mouse genotype. Comparable training and testing results were observed across all experimenters for all groups. Training and testing were conducted in a rectangle-shaped maze as described previously (Amodeo et al., 2017). The maze was divided into a start and choice areas by a guillotine door (Figure 2). A door opened up at the bottom center of the guillotine door. In the choice area, a piece extended from the back wall, which divided the area into two equally sized and distinct spatial locations. Both choice locations were adorned with distinct visuospatial cues attached to the back and sidewalls. In each location, a food well was centered and located 3 cm away from the back wall. The lighting in the maze was approximately 600 lumens. At the beginning of each training session, mice were placed into the start area. The start door was opened 1 min following placement into the holding chamber, allowing the mouse to freely navigate in the choice area and consume a 1/2 piece of Fruity Pebbles cereal (Post Foods, St. Louis, MO, USA) from each food well. After cereal pieces were consumed from both choice locations, the guillotine door was raised to allow a mouse to enter the start area. After a mouse had returned to the start area, the guillotine door was closed and the food wells re-baited. The start door was subsequently reopened to begin a new trial. This procedure was repeated until 15 min had elapsed. Mice were considered trained once they successfully completed seven trials in a 15 min session on each of 2 consecutive days. All mice trained reached this criterion. Mice were always trained for an odd number of trials on the last day of training. Which location a mouse chose first on a trial was recorded. Whichever location was chosen the fewer number of times became the “correct” location in the acquisition phase. This was to minimize initial learning being affected by a potential side bias.

#### 2.3.2. Acquisition, retention, and reversal learning

Acquisition and reversal learning each occurred in a single daily session across 2 consecutive days. In testing, only one of the two food wells was baited with a 1/2 piece of cereal in each trial. One location was designated as the “correct” spatial location and contained a 1/2 piece of cereal on 80% of trials. On the other 20% of trials, the “incorrect” location was baited with a 1/2 piece of cereal. The first two trials of each test always contained a food reinforcement in the “correct” arm. Criterion was achieved when a mouse chose the “correct” location on six consecutive trials. If a mouse chose a location with cereal, it was allowed to eat the cereal; the guillotine door was raised and subsequently lowered after a mouse returned to the start area. If a mouse chose a location with no cereal, it was allowed to navigate to the unbaited food well. Subsequently, the guillotine door was raised, allowing a mouse to return to the start area. If a mouse chose an unbaited food well, the baited food well was temporarily removed to prevent a mouse from quickly navigating over to the correct spatial location and obtain a cereal reinforcer after making an incorrect choice. Between trials, the choice area was cleaned with 2% ammonium chloride solution to minimize the use of odor cues. The retention and reversal learning tests were conducted the day after acquisition. A mouse first received a retention test as in previous experiments (Amodeo et al., 2017, 2018). In the retention test, a mouse was reinforced with 80% probability on trials for choosing the spatial location that was correct in acquisition. Criterion was achieved when a mouse successfully chose the “correct” spatial location (as in acquisition) on five out of six trials. Immediately after achieving retention criterion, reversal learning began. All aspects of the reversal learning test were identical to those in acquisition, except that the opposite spatial location was considered “correct” and reinforced with 80% probability. Criterion was met when a mouse made six consecutive correct choices. All mice tested achieved both acquisition and reversal learning criterion.

An error analysis of reversal learning was conducted as used previously in rodent models and patient-oriented ASD research (Brown et al., 2012; D’Cruz et al., 2013). The first reversal learning trial was not counted as a perseverative error, but served as initial negative feedback. On subsequent trials, if a mouse chose the previously correct spatial location, the choice was recorded as a perseverative error until a mouse first chose the new correct spatial location. After selecting the correct spatial location for the first time, all subsequent entries into the previously reinforced spatial location were scored as regressive errors.

In addition, an analysis was conducted to determine if KO mice compared to WT mice had differential sensitivity to reinforcement or lack of reinforcement on “correct” trials.

To determine this, trials were analyzed based on the outcome (reinforcement or no reinforcement) of each previous trial and shown as a ratio. For correct trials, win-stay ratios were determined by the number of times a subject received reinforcement in the “correct” compartment and then returned to the same compartment as that chosen on the previous trial, divided by the total number of reinforced trials for only the “correct” trials. The lose-shift ratio was determined by the number of times a subject reversed its choice after receiving no reinforcement in the “correct” spatial location on the previous trial, divided by the total non-reinforced trials for “correct” trials.

The sample size for each group was as follows: Male WT *n* = 10; Female WT *n* = 11, Male KO *n* = 12 and Female KO, *n* = 12.

#### 2.3.3. Elevated plus maze

A *different* set of mice were tested on the elevated plus maze than used in the spatial discrimination with probabilistic reinforcement test. Each mouse received a 10 min test on an elevated plus maze. The maze consisted of a gray acrylic radial arm maze that contained two closed arms with walls (40 cm in height) and two open arms. Remaining arms were inaccessible. The maze was elevated 50 cm from the floor and each arm was 21 cm long. Mice were introduced into the center area (9.5 cm × 9.5 cm) and allowed to freely explore the maze. The number of entries and time spent in the open arms, closed arms, and center were recorded using the ANY-maze software (Stoelting Inc., Wood Dale, IL, USA). An arm entry occurred when a mouse placed all four paws in open or closed arms.

The sample size for each group was as follows: Male WT, *n* = 10; Female WT, *n* = 10, Male KO, *n* = 12 and Female KO, *n* = 13.

#### 2.3.4. Reward conflict test

After completion of the elevated plus-maze test, mice were food restricted as described above for the spatial discrimination test. Training and testing occurred in the same maze used in the elevated plus maze test. Specifically, a mouse was placed in the central area and had access to four maze arms that each contained a back and side walls (20 cm height). Mice learned to obtain a half piece of cereal from each food well at end of each arm. After eating a cereal piece from each food well and returning to the center area the food wells were rebaited with cereal pieces. Training continued until a mouse completed a minimum of five trials in 15 min across two consecutive days. Testing occurred in the following session. The number of training trials varied across mice with an average of five training sessions required to achieve criterion.

In the reward conflict test, the maze was modified to have two open choice arms and two closed choice arms. The closed arms had walls 40 cm in height. Only the food wells in the open arms contained a half cereal piece. Each open arm foodwell contained a half cereal piece on each test trial. At the beginning of a test, a mouse was started in the center area and could enter either the closed arms or open arms. A choice was defined as entering all four paws in an arm. The open arms were designated as the correct arms and contained a half piece of cereal on each trial. If a mouse chose an open arm, it was allowed to eat a cereal piece and return to the center area marking the end of the trial. The foodwell was rebaited with a cereal after a mouse returned to the center area. If a mouse chose a closed arm, a mouse was allowed to locomote to the empty food well and return the center area, again marking the end of a trial. A correct choice was recorded if a mouse entered an open arm. This procedure was repeated until 30 min elapsed. Percent correct scores were calculated by dividing the number of open arm choices by the total number of choices multiplied by 100. Percent correct scores were calculated for three 10 min blocks. Testing was completed in a single test session.

### 2.4. Statistical analysis

For human behavioral results, statistical analyses were conducted using SPSS 19. Separate two-way analysis of variances (ANOVAs) were conducted to determine whether there was a significant difference in trials to criterion for acquisition, retention and reversal learning based on sex and/or diagnosis. Separate two-way ANOVAs were conducted to determine differences for perseverative errors, regressive errors, and lose-shift performance. We conducted planned *post hoc* comparisons to probe differences between sex and diagnosis using Fischer’s Least Significance Difference (LSD). To explore relationships between performance variables and demographic variables, we conducted Pearson correlations with age and IQ for each group. For FXS only, we explored relationships between performance variables and error rates from KiTAP tasks and clinical variables from parent-report measures. The statistical significance level was defined at *p* < 0.05.

For the mouse behavioral results, statistical analyses were conducted using GraphPad Prism. Separate two-way analysis of variances (ANOVAs) were conducted to determine whether there was a significant difference in trials to criterion for acquisition, retention and reversal learning based on sex and/or genotype. Separate two-way ANOVAs were conducted to determine differences for perseverative errors, regressive errors, win-stay and lose-shift performance. For the elevated plus-maze test, a two-way ANOVA determined whether there was a significant difference in open arm duration based on sex and/or genotype. For the reward conflict test, a three-way ANOVA with repeated measures determined whether percent correct scores differed across time, as well as based on sex and/or genotype. An analysis was conducted on the percent of open arm trials in which a mouse consumed a cereal piece. Fischer’s LSD tests were used to determine significant differences between groups. The statistical significance level was defined at *p* < 0.05.

## 3. Results

### 3.1. Human behavioral results

**FIGURE 3:**
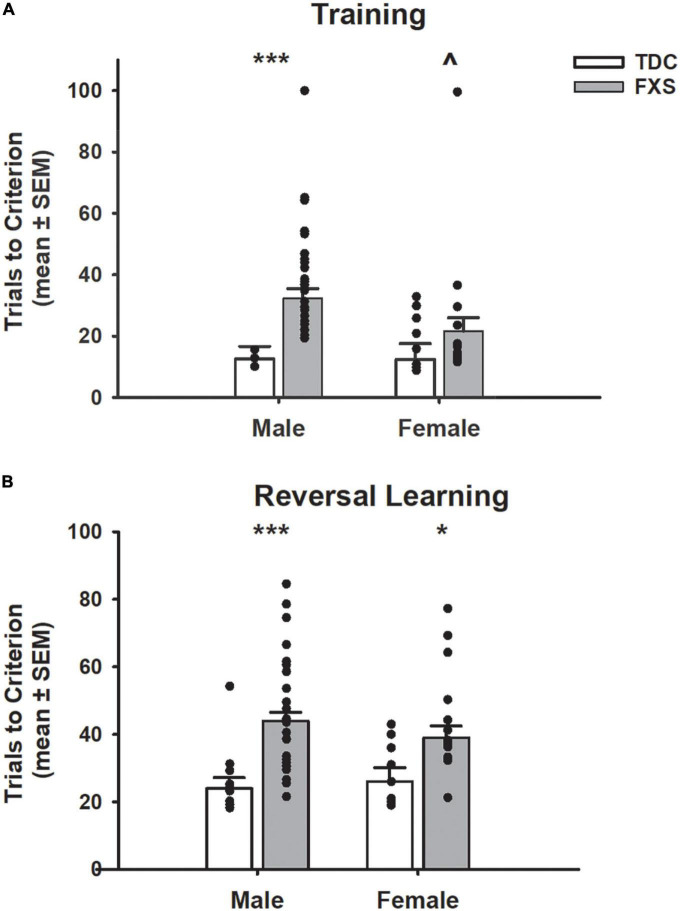
Trials to reach criterion on a spatial discrimination task (80/20 probabilistic learning procedure). **(A)** Mean (± SEM) trials to criterion on training task (no reversal). Males with fragile X syndrome (FXS), but not females with FXS required significantly more trials to criterion on acquisition than their TDC counterparts. Males with FXS also required more trials than females with FXS. **(B)** Mean (± SEM) trials to criterion on reversal learning. Males and females with FXS required more trials to reach criterion compared to that of control males and females. *p < 0.05 vs. TDC female; ***p < 0.001 vs. TDC male; ^∧^*p* < 0.05 vs. FXS male. Solid dot indicates individual participant data point.

In the training task (acquisition only; Figure 3A), FXS participants required more trials to reach criterion compared to TDC participants (*F*_1,85_ = 11.91, *p* = 0.001). Even though the group x sex interaction was not significant (*F*_1,85_ = 1.59, *p* = 0.211), males with FXS required a greater number of trials than their TDC counterparts (mean difference = 19.81; *t* = 3.92, *p* < 0.001), whereas there was not a significant effect for females (mean difference = 9.21; *t* = 1.36, *p* = 0.177). Of note, a significant difference emerged such that males with FXS required a greater number of trials during training compared to females with FXS (mean difference = 10.77; *t* = 2.03, *p* = 0.046).

During the probabilistic reversal learning task (Figure 3B), participants with FXS needed more total trials across acquisition and reversal phases to reach criterion than TDC (*F*_1,82_ = 23.90, *p* < 0.001). Group x sex interaction was not significant (*F*_1,82_ = 0.19, *p* = 0.664). Planned *post-hoc* tests revealed males (mean difference = 19.94, *t* = 4.91, *p* < 0.001) and females (mean difference = 13.00, *t* = 2.39, *p* = 0.019) with FXS participants required significantly more trials than their TDC counterparts. However, males and females with FXS did not differ from each other (*p* = 0.257). During the acquisition phase, the FXS group needed more trials to reach criterion than the TDC group (*F*_1,82_ = 10.46, *p* = 0.002), and this was found in both males (mean difference = 6.49, *t* = 2.78, *p* = 0.009) and females (mean difference = 6.49, *t* = 2.02, *p* = 0.047) with FXS. During the reversal phase, participants with FXS needed more trials to reach criterion (*F*_1,82_ = 18.28, *p* < 0.001). *Post-hoc* tests revealed males with FXS needed significantly more trials than control males (mean difference = 13.67, *t* = 4.80, *p* < 0.001), but females with FXS only needed marginally more trials than control females (mean difference = 6.51, *t* = 1.71, *p* = 0.088). Males and females with FXS did not differ from each other in terms of acquisition or reversal trials (*p*’s > 0.16).

**FIGURE 4:**
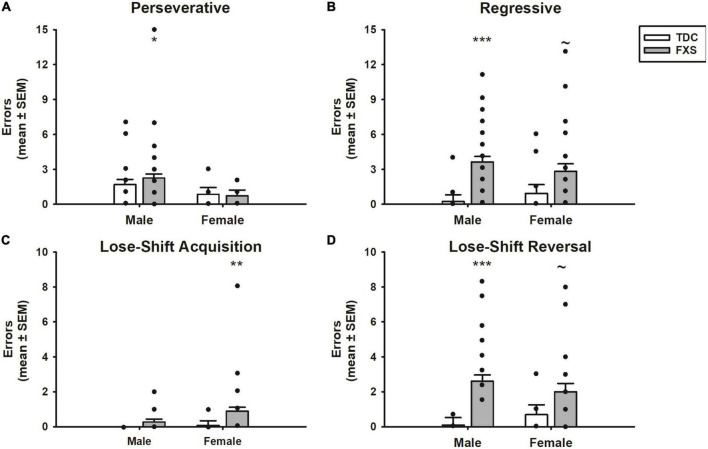
Error committed during reversal learning of a spatial discrimination (80/20 probabilistic learning procedure). **(A)** Mean (± SEM) perseverative errors during reversal learning. Fragile X syndrome (FXS) and TDC subjects did not differ in the number of perseverative errors. However, males with FXS made more perseverative errors than their TDC counterparts. **(B)** Mean (± SEM) regressive errors during reversal learning. FXS participants committed more regressive errors compared to controls during reversal learning. This finding was significant comparing male groups, but only trending when comparing female groups. **(C)** Mean (± SEM) lose-shift errors during acquisition. FXS participants made more lost-shift errors, which was driven by females. **(D)** Mean (± SEM) lose-shift errors during reversal learning. Males with FXS committed more lose-shift errors than TDC males, a finding that was only trending among females. *p < 0.05 vs. TDC males; **p < 0.05 vs. TDC females; ***p < 0.001 vs. TDC males; ∼*p* < 0.10 vs. TDC females. Solid dot indicates individual participant data point.

The different errors committed is illustrated in Figure 4. The number of perseverative errors during the reversal phase did not differ between FXS and TDC groups (*p* = 0.647). However, males made more perseverative errors than females across groups (*F*_1,82_ = 6.14, *p* = 0.015), which was driven by males with FXS committing more perseverative errors than females with FXS (mean difference = 1.82, *t* = 2.49, *p* = 0.015). However, the group x sex interaction was not significant nor was the difference between perseverative errors made by FXS males and TDC males (*p*’s > 0.474). In contrast, the number of regressive errors significantly differed between groups (*F*_1,82_ = 18.24, *p* < 0.001). The group x sex interaction was not significant (*F*_1,82_ = 1.45, *p* = 0.232). Although males committed more regressive errors during the reversal phase than their TDC counterpart (mean difference = 3.41, *t* = 4.54, *p* < 0.001), this difference approached significance among females (mean difference = 1.91, *t* = 1.90, *p* = 0.058). Number of regressive errors did not differ between males and females with FXS (*p* = 0.318).

To determine whether the groups differed in responsiveness after receiving a reinforcement or no reinforcement for a “correct” choice, we analyzed lose-shift errors during both acquisition and reversal phases. Participants with FXS made more lose-shift errors than controls during both acquisition (*F*_1,82_ = 6.16, *p* = 0.015) and reversal phases (*F*_1,82_ = 16.90, *p* < 0.001). Of note, during the acquisition phase, the group difference was driven by females with FXS (mean difference = 0.81, *t* = 2.33, *p* = 0.022), as males with FXS did not differ from controls (*p* = 0.304). Females with FXS also made more lose-shift errors during the acquisition phase than males with FXS (mean difference = 0.62, *t* = 2.19, *p* = 0.031). In contrast, during the reversal phase, males with FXS made more lose-shift errors than male controls (mean difference = 2.52, *t* = 4.48, *p* < 0.001), a group difference only approached significance in females with FXS (mean difference = 1.31, *t* = 1.75, *p* = 0.082). However, males and females with FXS did not differ from each other (*p* = 0.313).

Including age as covariate did not change any results. After accounting for Deviation IQ, groups no longer differed in total number of trials needed to reach criterion during either the training or probabilistic reversal learning tasks (*p*’s > 0.354). Number of errors also was no longer significantly different between groups after accounting for IQ (*p*’s > 0.219).

### 3.2. Correlations with demographic and clinical measures

In FXS, age did not relate to number of trials or errors (*p*’s > 0.225), with the exception of increased age was associated with more lose-shift errors during the acquisition phase (*r* = 0.31, *p* = 0.030), In TDC, increased age related to fewer total trials needed during the probabilistic reversal learning (*r* = −0.41, *p* = 0.014), and specifically during the acquisition phase (*r* = −0.35, *p* = 0.039). In FXS, lower Deviation IQ was related to more trials needed to reach criterion during the training task (*r* = −0.27, *p* = 0.049) and during probabilistic reversal learning (*r* = −0.29, *p* = 0.041) as well as more lose-shift errors (*r* = −0.32, *p* = 0.023) made during the reversal phase. In males with FXS, more lose-shift errors during the acquisition phase made were associated with higher deviation IQ (*r* = 0.40, *p* = 0.024). In females with FXS, more lose-shift errors during the acquisition phase made were associated with increased age (*r* = 0.55, *p* = 0.017), whereas increased trials needs to reach criterion was associated with lower deviation IQ (*r* = −0.65, *p* = 0.004), specifically during the acquisition phase (*r* = −0.55, *p* = 0.019). In TDC, lower deviation IQ also was related to more lose-shift errors (*r* = −0.34, *p* = 0.043).

A further examination of error type during probabilistic reversal learning and clinical measures was conducted. Important to note, perseverative errors were made only by a subset of participants and usually not in the same participant who committed regressive errors, consistent with the fact that the number of perseverative and regressive errors made did not relate to each other in males with FXS (*r* = 0.12, *p* = 0.51). Perseverative errors did not relate to any clinical measures used in the study. A follow-up analysis was conducted to compare fully methylated, full mutation males and mosaics males. Full mutation males tended to make more perseverative errors (3.2 ± 3.9) than mosaic males (1.2 ± 1.2), though this did not reach significance (*p* = 0.133). Also, increased perseverative errors made related to more severe restricted, repetitive behaviors in full mutation males (ρ = 0.73, *p* = 0.040), but not in mosaic males (ρ = −0.23, *p* = 0.525).

In FXS, committing more regressive errors was related to more severe stereotyped behaviors on the ABC-C (ρ = 0.32, *p* = 0.025). Needing more trials to reach criterion during the acquisition phase related to more severe stereotyped behavior on the RBS-R (ρ = 0.30, *p* = 0.049) and lower social awareness on the SRS-II (ρ = 0.42, *p* = 0.016). Increased lose-shift errors and trials during the reversal phase related to reduced sensory seeking behavior (error: ρ = −0.38, *p* = 0.024; trial: (ρ = 0.34, *p* = 0.045).

**FIGURE 5:**
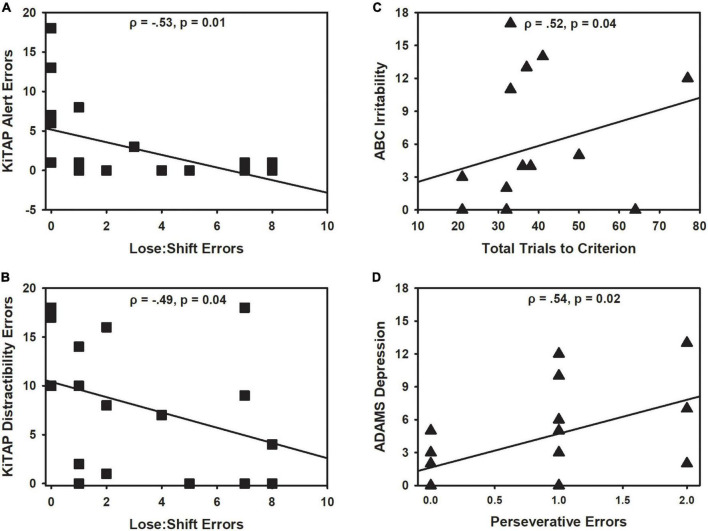
Correlations with clinical data for males and females with fragile X syndrome (FXS). **(A)** Increased lose:shift errors during the reversal phase related to fewer anticipation errors during KiTAP Alert task in FXS males (black square). **(B)** Likewise, lose:shift errors during the reversal phase related to fewer errors during KiTAP Distractibility task in FXS males. **(C)** In contrast, increased total trials needed to reach criterion related to more severe ABC irritability ratings in FXS females (black triangle). **(D)** Increased perseverative errors related to more severe ADAMS ratings in FXS females as well. Spearman correlation and *p*-values provided for each graph.

In males with FXS (*n* = 19), increase lose-shift errors made and reversal trials needed to reach criterion related to fewer omission (error: ρ = −0.54, *p* = 0.012, Figure 5A; trial: ρ = −0.50, *p* = 0.020) and anticipation errors (error: ρ = −0.53, *p* = 0.013; trial: ρ = −0.53, *p* = 0.014) during KiTAP Alertness. Making more lose-shift errors during probabilistic reversal learning also related to making fewer errors during KiTAP Distractibility (ρ = −0.49, *p* = 0.039, Figure 5B) and Go/NoGo tasks (ρ = −0.47, *p* = 0.040), but reversal errors did not relate on those during the KiTAP Flexibility task (*p*’s > 0.473). In contrast, none of these relationships were significant in females, but this may be due to smaller sample size with both datasets (*n* = 7).

In females with FXS, increased lose-shift errors (ρ = −0.35, *p* = 0.048) and trials during the reversal phase (ρ = −0.38, *p* = 0.032) related to less severe speech abnormalities on the ABC-C. In contrast, in females increased number of total trials to reach criterion related to increased irritability on the ABC-C (ρ = 0.52, *p* = 0.040; Figure 5C). In addition, increased perseverative errors made related to more severe depressive symptoms on the ADAMS (ρ = 0.55, *p* = 0.024; Figure 5D) and increased number of acquisition trials related to more severe OCD-like symptoms on the ADAMS (ρ = 0.62, *p* = 0.007).

### 3.3. Mouse behavioral results

#### 3.3.1. Spatial acquisition, retention, and reversal learning

**FIGURE 6:**
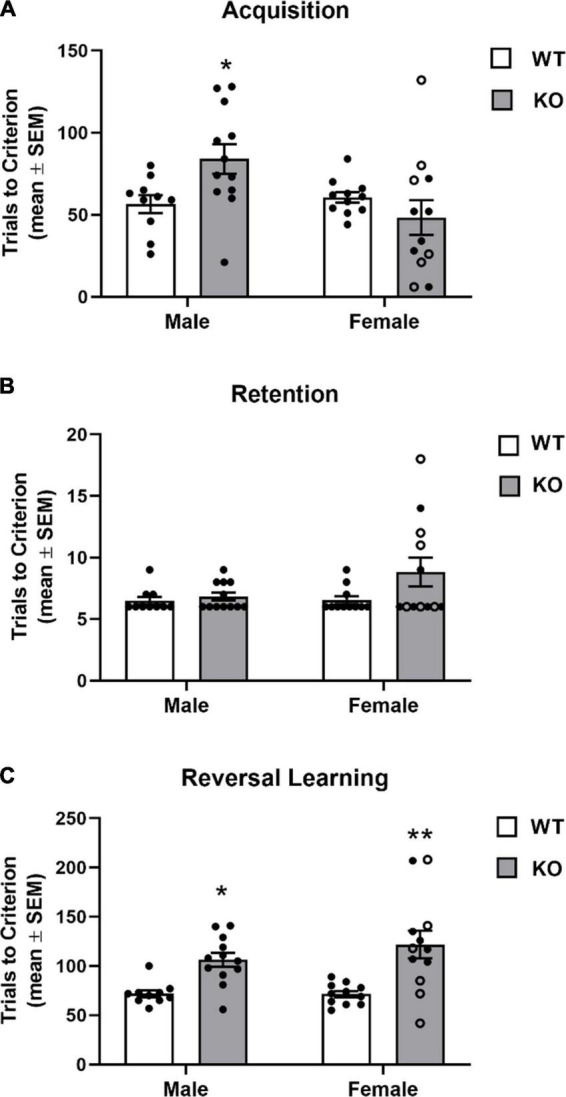
Acquisition and reversal learning of a spatial discrimination task (80/20 probabilistic learning). **(A)** Mean (± SEM) trials to criterion on acquisition. Male *Fmr1*-knockout (KO) mice, but not female *Fmr1*-KO mice required significantly more trials to criterion on acquisition than wildtype (WT) male and female mice. **(B)** Mean (± SEM) trials to criterion on retention. WT and *Fmr1*-KO mice required similar number of trials to criterion in retention. **(C)** Mean (± SEM) trials to criterion on reversal learning. Male and female *Fmr1*-KO mice required more trials to reach criterion compared to that of WT male and female mice. *p < 0.05 vs. WT male and female mice; **p < 0.01 vs. WT male and female mice. In the female *Fmr1-* KO group, •, heterozygous mice and ∘, homozygous mice.

The findings from the spatial discrimination are shown in Figure 6. WT mice (female and male) achieved acquisition criterion in approximately 60 trials. Female KO mice exhibited a similar learning rate as WT mice. Although for the female KO group there was a wider range of trials to criterion across mice with a subset exhibiting reduced trials to criterion on acquisition while another subset of mice requiring a greater number of trials to criterion than WT. In contrast, the male KO group more consistently required more trials to criterion than WT obtaining criterion in around 85 trials. Analysis of the acquisition results revealed there was not a significant main effect for genotype (*F*_1,41_ = 0.92, *p* = 0.34) or sex (*F*_1,41_ = 3.91, *p* = 0.07), but there was a significant genotype x sex interaction (*F*_1,41_ = 6.19, *p* = 0.02). A *post hoc* analyses indicated that male KO mice required significantly more trials in acquisition compared to that of male and female WT mice (*p*’s < 0.05).

In the retention test, all groups required a similar amount of trials to criterion (see Figure 6B). The analysis indicated there was no significant effect for genotype (*F*_1,41_ = 3.34, *p* = 0.08), sex (*F*_1,41_ = 2.00, *p* = 0.17) or genotype x sex interaction (*F*_1,41_ = 1.82, *p* = 0.19). Despite no significant main effects or interaction, somewhat similar to that observed in acquisition, a subset of female KO mice required a greater number of trials during the retention test (10–20 trials).

In reversal learning, WT mice obtained criterion in approximately 70 trials. In contrast, both KO groups required over 100 trials to achieve criterion (Figure 6C). There was a significant genotype effect (*F*_1,41_ = 23.51, *p* < 0.0001). There was not a significant effect for sex (*F*_1,41_ = 0.74, *p* = 0.39) nor a significant genotype x sex interaction (*F*_1,41_ = 0.84, *p* = 0.37).

**FIGURE 7:**
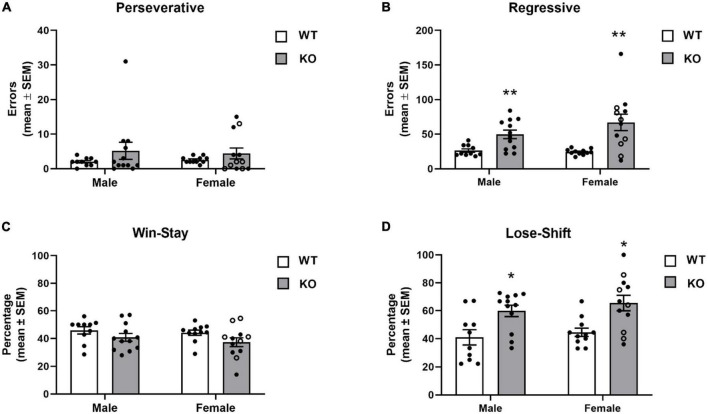
Error committed during reversal learning of a spatial discrimination (80/20 probabilistic learning procedure). **(A)** Mean (± SEM) perseverative errors during reversal learning. Wildtype (WT) and *Fmr1*-knockout (KO) mice did not differ in the number of perseverative errors. **(B)** Mean (± SEM) regressive errors during reversal learning. Male and female *Fmr1*-KO mice committed more regressive errors compared to that of WT male and female during reversal learning. **(C)** Mean (± SEM) percentage win-stay probabilities during reversal learning. *Fmr1*-KO mice had lower win-stay probabilities than WT mice. **(D)** Mean (± SEM) percentage lose-shift probabilities during reversal learning. Both male and female *Fmr1*-KO mice had higher lose-shift probabilities than WT male and female mice. *p < 0.05 vs. WT male and female mice; **p < 0.01 vs. WT male and female mice. In the female *Fmr1-* KO group, •, heterozygous mice and ∘, homozygous mice.

The different errors committed is illustrated in Figure 7. In general, mice displayed a low number of perseverative errors. However, in both male and female KO mice, a subset of mice repeatedly chose the previously “correct” spatial location before first choosing the new “correct” spatial location as reflected by a higher number of perseverative errors. The analysis indicated no significant effects for genotype (*F*_1,41_ = 2.45, *p* = 0.13), sex (*F*_1,41_ = 0.004, *p* = 0.95) or genotype x sex interaction (*F*_1,41_ = 0.16, *p* = 0.69). In contrast to perseverative errors, analysis of regressive errors showed that there was a significant effect for genotype (*F*_1,41_ = 20.51, *p* < 0.0001). *Post-hoc* tests revealed that male and female KO mice committed significantly more regressive errors during reversal learning than both male and female WT mice (*p*’s < 0.01). There was not a significant effect for sex (*F*_1,41_ = 1.11, *p* = 0.30) or genotype x sex interaction (*F*_1,41_ = 1.71, *p* = 0.20).

To determine whether the mouse strains differed in responsiveness after receiving a reinforcement or no reinforcement for a “correct” choice, win-stay and lose-shift probabilities were analyzed (Figures 7C, D). WT mice had win-stay probabilities around 45%. KO mice had lower win-stay probabilities. The analysis indicated the difference in win-stay probabilities lead to a significant genotype effect (*F*_1,41_ = 4.67, *p* = 0.04). *Post hoc* analyses indicated no significant differences between the groups. There was not a significant effect for sex (*F*_1,41_ = 0.92, *p* = 0.34) or genotype x sex interaction (*F*_1,41_ = 0.11, *p* = 0.78). Analysis of the lose-shift probabilities indicated a significant genotype effect (*F*_1,41_ = 18.33, *p* = 0.0001) reflecting that both male and female KO mice had significantly greater lose-shift probabilities than WT (*p*’s < 0.05). There was not a significant effect for sex (*F*_1,41_ = 0.99, *p* = 0.32) or genotype x sex interaction (*F*_1,41_ = 0.05, *p* = 0.83).

#### 3.3.2. Elevated plus maze

**FIGURE 8:**
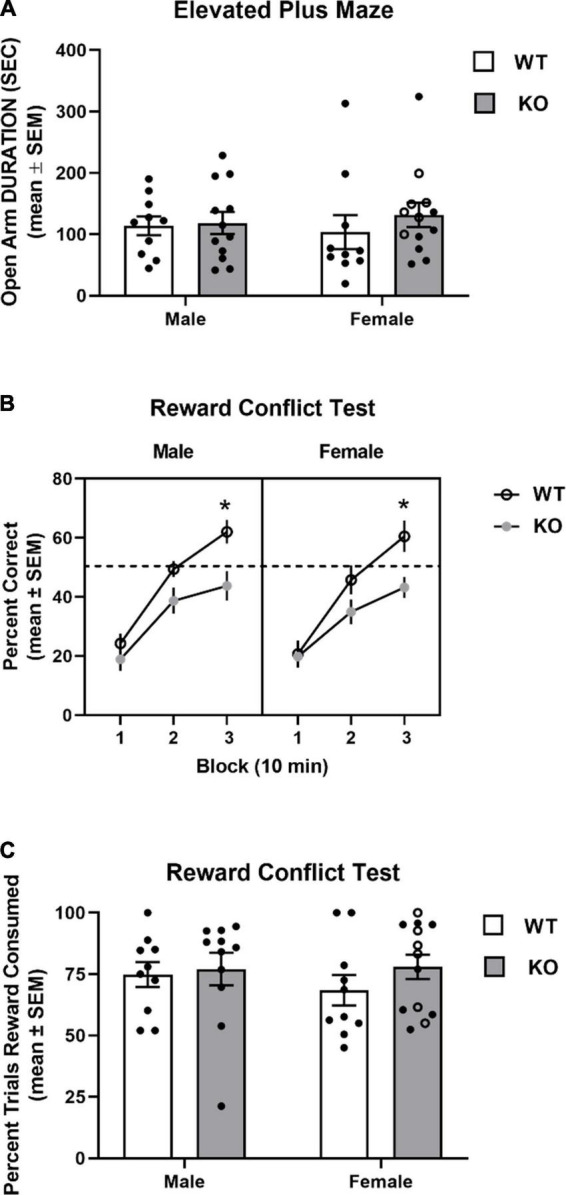
Performance in elevated plus maze and reward conflict test. **(A)** Mean (± SEM) duration in the open arms in the elevated plus maze test. Wildtype (WT) and *Fmr1*-knockout (KO) mice did not differ in open arm duration. **(B)** Mean (± SEM) percent correct across three 10 min blocks in reward conflict test. WT and *Fmr1*-KO mice exhibited similar performance during the first block of testing with male and female WT mice exhibiting greater performance than *Fmr1*-KO mice during the second and third blocks. *p < 0.05 vs. WT male and female mice. **(C)** Mean (± SEM) percent of open arm trials cereal reward was consumed. WT and *Fmr1*-KO mice did not differ in percent of open arm trials cereal reward was consumed. In the female *Fmr1-* KO group, •, heterozygous mice and ∘, homozygous mice.

Mice from all groups exhibited similar time spent in the open arms of the elevated plus maze (see Figure 8A). A two-way ANOVA analysis revealed that there was no significant effect for genotype (*F*_1,41_ = 0.62, *p* = 0.43), sex (*F*_1,41_ = 0.005, *p* = 0.94), nor genotype x sex interaction (*F*_1,41_ = 0.33, *p* = 0.57).

#### 3.3.3. Reward conflict maze

The results from the reward conflict test are shown in Figure 8B. All groups achieved approximately 20% accuracy during the first 10 min block. Subsequently, WT mice approached chance performance (50%) in the second block and had around 60% accuracy in the last block. In contrast, KO mice had 35–40% by the second block and around 45% accuracy by the third block. A three-way ANOVA with repeated measures indicated a significant block effect (*F*_1.6,67_ = 140.4, *p* < 0.0001), reflecting the improved learning across blocks. There was also a significant genotype effect (*F*_1,41_ = 8.79, *p* = 0.005) indicating that WT mice had greater performance in the test compared to that of KO mice. There was also a significant block x genotype effect (*F*_2,82_ = 7.36, *p* = 0.001). *Post hoc* analyses revealed that both KO groups were not significantly different from WT groups in blocks 1 and 2, but had significantly decreased accuracy in block 3 compared to that of male and female WT mice (*p*’s < 0.05).

Mice did not consume a cereal piece on all trials in which the open arm was chosen. The percent of open arm trials in which a mouse consumed a cereal reward was determined for mice in all groups. Mice from all groups consumed a cereal piece when choosing an open arm around 75% of trials (see Figure 8C). An analysis of percent of open trials a cereal piece was consumed revealed that there was a not a significant effect for genotype (*F*_1,40_ = 1.05, *p* = 0.31) or sex (*F*_1,40_ = 0.23, *p* = 0.64), nor a significant genotype x sex interaction (*F*_1,40_ = 0.40, *p* = 0.53).

## 4. Discussion

The present study is the first of its kind taking a translational approach to characterize learning and cognitive flexibility deficits in both individuals with FXS and *Fmr1*-KO mice by employing a similar spatial discrimination test with probabilistic reinforcement. The cross-species investigation found both similarities and differences between individuals with FXS and *Fmr1*-KO mice and in performance on the probabilistic learning and reversal learning tests. In males with FXS and male *Fmr1*-KO mice there was a striking parallel in that both groups exhibited an initial probabilistic learning deficit as well as a probabilistic reversal learning impairment. In contrast, females of both species demonstrated similar probabilistic reversal learning deficits. Taken together, the findings suggest that the spatial discrimination with probabilistic reinforcement test can be effectively used in a bidirectional translational manner to understand cognitive flexibility deficits in neurodevelopmental disorders, like FXS, which is critical for facilitating treatment development.

A major advantage of the spatial discrimination test as used in mice is that the initial learning and reversal learning are each completed in a single session that is more similar to how testing is applied in human subjects. This contrasts past studies involving *Fmr1*-KO mice that employed tests in which the initial learning and reversal learning phases required several sessions, i.e., Morris water maze (Kooy et al., 1996; D’Hooge et al., 1997; Eadie et al., 2009; Boda et al., 2014; Leach et al., 2016). The completion of initial learning and reversal learning in a single session not only approximates the procedure carried out in humans, but also removes ambiguity that a deficit or lack of a deficit may be related to memory consolidation processes. While the spatial discrimination and reversal test used in humans and mice had similarities there were also some distinct differences. Acquisition and reversal learning in humans was completed in a single session while in mice acquisition occurred in one daily session followed by retention and reversal learning in a session 24 h later. In addition, mice were food-restricted and received a sweetened cereal piece when making a correct choice while humans when making a correct choice received visual feedback with an artificial monetary reward. Despite these procedural differences there was strong similarities in how *Fmr1*-KO mice and FXS subjects performed on these tests.

Another unique feature of the probabilistic reversal learning test to study cognitive flexibility compared to past measures in *Fmr1*-KO mice and FXS participants is that the present investigation used probabilistic reinforcement as opposed to deterministic outcomes, e.g., one choice always correct, one choice always incorrect. Probabilistic learning tests are considered to be more ecologically relevant of choices that humans and other mammalian species face in daily living as it adds a feature of unexpected non-reinforcement. Examination of learning and reversal learning with probabilistic reinforcement may have a distinct advantage to tests with deterministic outcomes because previous investigations with *Fmr1*-KO mice using discrimination tests with deterministic outcomes have led to variable results with reports of no initial learning or reversal learning deficit, a transient deficit on learning or reversal learning or a deficit in both phases (Kooy et al., 1996; D’Hooge et al., 1997; Eadie et al., 2009; Casten et al., 2011; Dickson et al., 2013; Boda et al., 2014; Leach et al., 2016). In humans, examination of cognitive flexibility in FXS have relied on tasks like dimensional card sorting which require multiple aspects of higher-level cognition, like working memory and inhibitory control, and thus are less specific to cognitive flexibility [for review see Schmitt et al., 2019b]. Thus, the probabilistic learning and reversal learning tests may produce a more robust phenotype in *Fmr1*-KO mice that can advance understanding and treating cognitive deficits in FXS. Although our analyses did not correct for multiple comparisons, which will be important in future replication studies, we believe our findings emphasize the promise of the probabilistic learning and reversal learning tests in translational cross-species research in FXS.

In female mice and humans, the probabilistic learning and reversal learning tests showed not only a more intermediate phenotype, but also a more variable phenotype. However, this finding is expected given females with FXS are obligate mosaics (one X chromosome still produces FMRP) and random X chromosome inactivation (Bartholomay et al., 2019). There was not a significant effect on probabilistic learning in female *Fmr1*-KO mice, but a subset of female *Fmr1*-KO mice required 30 or more trials than the mean to achieve criterion. In general, there was greater variance observed in the female *Fmr1*-KO group with a subset of mice also exhibiting facilitated learning. In reversal learning, female *Fmr1*-KO mice were significantly impaired similar to that observed with male *Fmr1*-KO mice. Analogous findings were observed in females with FXS as they did not differ from female controls on probabilistic training though a small subset of females required 10 or more trials than the mean to achieve criterion. During reversal learning, females with FXS, like males, needed more trials than controls, but again this seems to be driven by a small subset of females rather than a near universal effect in males with FXS. Despite the potential clinical relevance of these findings, some caution is needed when interpreting these findings since many of these sex differences only emerged when examining planned *post hoc* analyses that were not correct for multiple comparisons.

The current task also allows for the analysis of different error type to further identify what processes are altered that contributed to a reversal learning impairment, which cannot be examined and/or not always reported in studies with rodents or humans (Robbins, 2017; Schmitt et al., 2019b). Our error analysis ascertained whether a reversal learning deficit emerged due to a deficit in initially inhibiting the previously learned choice pattern (i.e., perseverative) and/or maintaining the new choice pattern once selected (i.e., regressive). Across mice and humans, FMRP deletion led to a significant increase in regressive errors. The increase in regressive errors was observed in both males and females with a FMRP deletion, although this finding only approached significance in females with FXS, similar to that observed for probabilistic reversal learning. One parsimonious explanation for these more limited effects in female FXS participants is that they are obligate mosaics and thus still produce some FMRP that may mute the overall cognitive flexibility deficit. However, the general effect of an increase in regressive errors suggest that *Fmr1*-KO mice and FXS individuals could initially inhibit the previously learned choice pattern and switch to the new, correct choice pattern, but were impaired in reliably executing the new choice pattern. The increase in regressive errors, but not perseverative errors, observed in *Fmr1*-KO mice is comparable to the error pattern observed in male *Fmr1*-KO mice on reversal of a visual discrimination test (Dickson et al., 2013). However, because males with FXS demonstrated increased perseverative errors compared to females with FXS, findings differ from those reported in ASD participants using the same test (D’Cruz et al., 2013). Thus, committing perseverative errors, or the impaired ability to initially inhibit a previously learned choice pattern, may be a behavior specific to males with FXS. Perseveration errors during tasks of cognitive flexibility previously have been reported in males with FXS, and even seem specific to this patient population compared to others associated with intellectual disability, like Down Syndrome (Cornish et al., 2001; Kogan et al., 2009).

In participants with FXS, perseverative errors were made only by a subset of subjects. Separating out males with FXS into full mutation and mosaics revealed that full mutation males committed approximately three times the number of perseverative errors compared to that of mosaic males and perseverative errors in the full mutation group correlated with more severe restricted, repetitive behaviors. Together, this suggests that committing perseverative errors may be specific to a subset of males with FXS who have broader impairments failure to disengage from a previously reinforced stimulus when it is no longer rewarded.

On the other hand, the elevated number of regressive errors may result from *Fmr1*-KO mice and FXS individuals having a greater sensitivity to negative reinforcement/feedback. This is because both groups displayed a significant increase in lose-shift errors such that when they chose a “correct” location followed by negative feedback, they were more likely than controls to switch to the previously “correct” location on the subsequent trial. The optimal strategy is to ignore the occasional negative feedback (lack of reinforcement) and maintain selecting the same choice. Trials when a “correct” choice is not reinforced may be particularly challenging to maintain the current choice pattern as earlier studies with FXS males that range in age from early childhood to adulthood have reported deficits in inhibiting distractor information (Munir et al., 2000; Cornish et al., 2007; Hooper et al., 2008). Of note, we found that making lose-shift errors related to fewer errors during other computerized performance-based tasks of alertness, distractibility, and inhibition in males with FXS. Although unexpected non-reinforcement disrupts ability to follow current task instructions, exaggerated reward sensitivity may help guide future and correct performance in predictable situations, as demonstrated in KiTAP tasks when provide deterministic feedback. Thus, FMRP deficiency exaggerating reward sensitivity, as previously reported in FXS (Fish et al., 2013), may in fact be adaptive in daily lives though future work is needed to confirm this assertion.

The various impairments identified in probabilistic reversal learning from both *Fmr1*-KO mice and FXS individuals also have implications on what brain circuitry may be altered underlying the cognitive flexibility deficits. Specifically, there is extensive evidence that frontal-striatal circuits across mammalian species support cognitive flexibility (Stelzel et al., 2013; Oemisch et al., 2019; Williams and Chritakou, 2021) and that this circuitry may be altered in multiple disorders that exhibit cognitive inflexibility (Marsh et al., 2014; D’Cruz et al., 2016; Langley et al., 2021). Both preclinical and clinical studies suggest that the striatum is critical for reliably executing a new choice pattern during reversal learning (Haluk and Floresco, 2009; Hill et al., 2015; Sleezer and Hayden, 2016; Grospe et al., 2018) and treatments that improve probabilistic reversal learning can rescue probabilistic reversal learning deficits in mouse models of autism when infused directly into the dorsomedial striatum by reducing regressive errors (Amodeo et al., 2017; Athnaiel et al., 2022). Further, brain imaging studies in humans indicate that typically developing subjects exhibit significant activation in frontal cortex and ventral striatum when outcomes are uncertain in a reversal learning test while ASD individuals do not display activation in this brain circuitry under the same test conditions (D’Cruz et al., 2011, 2016). Structural imaging studies have demonstrated alterations in white matter tract circuity and volumes within dorsal–prefrontal areas (including the caudate) in individuals with FXS (Peng et al., 2014). In the context of the current study, abnormalities within orbitofrontal cortex may alter reward processing systems (i.e., lose-shift errors), and abnormalities within dorsolateral prefrontal cortex may help maintain repetitive behavior (i.e., perseverative or regressive errors). In addition, altered function in the striatum of both *Fmr1*-KO mice and FXS individuals also may have contributed significantly to the increase in lose-shift and regressive errors that resulted in a probabilistic reversal learning deficit. However, no functional imaging or electrophysiological study using a task of cognitive flexibility has been completed in FXS. Overall, the error pattern during probabilistic reversal learning is consistent with results in *Fmr1*-KO mice and FXS individuals showing alterations in the striatum or caudate and prefrontal cortical regions (Centonze et al., 2008; Hoeft et al., 2010).

Past investigations examining cognitive function in *Fmr1*-KO mice and FXS individuals have predominantly been limited to male subjects. The current investigation characterized females in both mouse and human subjects. With mice, a combination of heterozygous and homozygous females were tested. There were not differences between heterozygous and homozygous female mice on any of the behavioral measures and thus they were combined as a single group. However, as noted above, female *Fmr1*-KO mice often showed greater variability on the various measures with a subset having higher scores on measures such as acquisition, retention and perseveration despite there not being a significant group effect. The subset of mice having high scores on these measures were not always the same mice and were a mix of heterozygous and homozygous females. One possibility is that this subset of females captured on the probabilistic learning and reversal learning tests more accurately models the real-world heterogeneity observed in females with FXS. This is a critical point because failure of pre-clinical drug trials to translate efficacy into human trials has been thought to result from poor rodent models of the human condition and its variability (Budimirovic et al., 2017; Erickson et al., 2017; Berry-Kravis et al., 2018). In addition, future studies that investigate heterozygous and homozygous female *Fmr1* mice on a broader range of behavioral tests will be critical for better understanding the similarities and differences in phenotype between the two different genotypes.

It also is important to note that in females with FXS, we found that increased number of trials to reach criterion and errors made related to more severe psychiatric features of irritability, depression, and OCD. This is in direct contrast to correlational findings in males with FXS demonstrating probabilistic reversal learning errors related to broader deficits across multiple areas of executive function. To the best of our knowledge, this is the first time that a study has demonstrated a significant relationship between cognitive flexibility deficits and psychiatric features in females with FXS despite both being regularly reported clinically and in the literature. Although we are not able to determine the direction of this relationship, it does suggest that these phenotypes may co-occur within specific females rather than both be present, but in different females with FXS. In our recent examination of female premutation carriers (PMCs), although we reported that psychiatric symptoms and executive function deficits defined two separate subgroups of PMCs, but that females with more severe anxiety and depressive symptoms seemed to be most at risk for co-occurring executive function deficits (Schmitt et al., 2021). This is not only important to think about in terms of underlying mechanisms that may maintain cognitive and psychiatric features, but also in terms of treatment planning. Future studies are needed to determine how underlying pathology of *FMR1* as well as environmental factors including the stress of having a genetic disorder contribute to cognitive flexibility deficits and psychiatric features in females with FXS.

In addition to cognitive flexibility impairments involving inhibiting a learned response and switching to a different or new, learned response pattern, FXS individuals display deficits in withholding prepotent responses when contextually inappropriate (Munir et al., 2000; Scerif et al., 2005; Tonnsen et al., 2015). In an attempt to model this in mice, the reward conflict test was developed in which mice are required to overcome their prepotent bias to avoid open arms in an elevated maze and enter open arms to obtain a cereal reward. Both WT and KO mice exhibited a comparable response bias toward avoiding the open arms in the first test block. In subsequent blocks, WT mice displayed a greater ability to inhibit the prepotent response and enter the open arms in the majority of trials by the last test block. The impairment in the reward conflict test observed in male and female *Fmr1*-KO mice cannot simply be explained by a more general increase in “anxiety” or decrease in motivation because *Fmr1*-KO mice showed similar performance as WT mice in the elevated-plus maze test and *Fmr1*-KO mice were just as likely to consume a cereal piece when they entered an open arm as WT mice. Instead the pattern of results suggest that *Fmr1*-KO mice are slow to learn in withholding a prepotent response in order to obtain positive reinforcement. This may help account for our finding in males with FXS showing a relationship between lose-shift errors made and errors on the KiTAP Go/Nogo task of prepotent inhibition. Further the reward conflict test offers another paradigm to be used in preclinical research to develop treatments in order to improve cognitive flexibility when conditions require inhibiting a prepotent response.

Overall, the phenotype was remarkably similar in the probabilistic reversal learning test for *Fmr1*-KO mice and FXS individuals, particularly among males. This suggests that this test may be a useful translation approach to understand the neuropatholophysiology contributing to cognitive and behavioral flexibility deficits in FXS as well as speed development of novel treatments to improve lives of individuals with FXS.

## Data availability statement

The original contributions presented in this study are included in the article/Supplementary material, further inquiries can be directed to the corresponding author.

## Ethics statement

The studies involving human participants were reviewed and approved by Cincinnati Children’s Hospital Medical Center Institutional Review Board. Written informed consent to participate in this study was provided by the participants’ legal guardian/next of kin. The animal study was reviewed and approved by University of Illinois Chicago Institutional Animal Care Use Committee.

## Author contributions

LS participated in the conceptualization of the study, analyzed and interpreted human data, and assisted in drafting manuscript. JS and CE assisted in the conceptualization of the study and edited the manuscript. AA participated in the conceptualization of the mouse studies, collected, analyzed, and interpreted mouse data along with drafting the manuscript. JD and MP assisted in the collection and analysis of the mouse data. JL and MR participated in the conceptualization of the mouse studies, analyzed and interpreted mouse data along with drafting the manuscript. All authors contributed to the article and approved the submitted version.
