# Supplementary material for: Parallel learning and cognitive flexibility impairments between Fmr1 knockout mice and individuals with fragile X syndrome
Source: Front Behav Neurosci. 2023 Jan 5;16:1074682. doi: 10.3389/fnbeh.2022.1074682 (PMC9849779; doi:10.3389/fnbeh.2022.1074682)
Supplement: Supplementary file 1 [file Data_Sheet_1.PDF]

# Supplementary Material

Supplemental Table 1. Psychotropic medication information for FXS participants.

| Psychotropic Medications | FXS |
|---|---|
| **No Psychotropic Medications** | 19 (35%) |
| **Prescribed Psychiatric Medications** | 34 (63%) |
| SSRI | 20 (37%) |
| Other Antidepressants | 1 (2%) |
| Atypical Antipsychotics | 16 (30%) |
| Benzodiazepines | 6 (11%) |
| Anticonvulsant | 3 (6%) |
| Stimulant | 7 (13%) |
| Non-Stimulant Medication for ADHD | 6 (11%) |
| Sleep Aid | 2 (4%) |
| **Multiple Psychotropic Medications** | 18 (33%) |
| **Medication Information Unavailable** | 1 (2%) |
